# Redox-Regulated Pathway of Tyrosine Phosphorylation Underlies NF-κB Induction by an Atypical Pathway Independent of the 26S Proteasome

**DOI:** 10.3390/biom5010095

**Published:** 2015-02-09

**Authors:** Sarah Cullen, Subramaniam Ponnappan, Usha Ponnappan

**Affiliations:** 1 Department of Microbiology and Immunology, University of Arkansas for Medical Sciences, Little Rock, AR 72205, USA; E-Mail: cullensarahjane@gmail.com; 2 Department of Geriatrics, University of Arkansas for Medical Sciences, Little Rock, AR 72205, USA; E-Mail: SPonnappan@uams.edu

**Keywords:** IκBα, NF-κB, proteasome, phosphotyrosine, pervanadate, oxidative stress, hypoxia/reoxygenation

## Abstract

Alternative redox stimuli such as pervanadate or hypoxia/reoxygenation, induce transcription factor NF-κB by phospho-tyrosine-dependent and proteasome-independent mechanisms. While considerable attention has been paid to the absence of proteasomal regulation of tyrosine phosphorylated IκBα, there is a paucity of information regarding proteasomal regulation of signaling events distinct from tyrosine phosphorylation of IκBα. To delineate roles for the ubiquitin-proteasome pathway in the phospho-tyrosine dependent mechanism of NF-κB induction, we employed the proteasome inhibitor, Aclacinomycin, and the phosphotyrosine phosphatase inhibitor, pervanadate (PV). Results from these studies demonstrate that phospho-IκBα (Tyr-42) is not subject to proteasomal degradation in a murine stromal epithelial cell line, confirming results previously reported. Correspondingly, proteasome inhibition had no discernable effect on the key signaling intermediaries, Src and ERK1/2, involved in the phospho-tyrosine mechanisms regulating PV-mediated activation of NF-κB. Consistent with previous reports, a significant redox imbalance leading to the activation of tyrosine kinases, as occurs with pervanadate, is required for the induction of NF-κB. Strikingly, our studies demonstrate that proteasome inhibition can potentiate oxidative stress associated with PV-stimulation without impacting kinase activation, however, other cellular implications for this increase in intracellular oxidation remain to be fully delineated.

## 1. Introduction

The nuclear factor-κB (NF-κB) family of transcription factors is comprised of evolutionarily conserved and structurally-related interacting proteins that bind to DNA. NF-κB plays a crucial role in the transcriptional regulation of genes involved in controlling cell proliferation, differentiation, apoptosis, inflammation, and stress responses, in addition to other biological processes. Consistent with its role in regulating the immune response, abnormal NF-κB regulation has been linked to cancer, aging, inflammatory and autoimmune diseases, septic shock, viral infections, and improper immune development. Several activating mechanisms for NF-κB have been described, each of which is inducible in a cell and stimuli-specific manner.

Activation of NF-κB by proinflammatory cytokines belonging to the tumor necrosis factor (TNF) and interleukin (IL)-families is termed the canonical pathway, whereas activation of a specific Ser/Thr-specific IκB kinase (IKK) signalosome complex, active in B-cells, is a second and alternative pathway. The third group of mechanisms, collectively termed “atypical pathways”, comprises chemical and physiological stress factors [1], as well as oxidative [2], genotoxic [3], and organelle stress [4]. Despite the diversity amongst these signaling pathways, there are considerable similarities in terms of the underlying mechanisms associated with NF-κB induction. For example, phosphorylation-dependent inactivation of IκB proteins, such as IκBα or p100, dominates as the prevailing mechanism for the vast majority of these NF-κB signaling pathways [5]. Such a phosphorylation pathway has been extensively characterized for IκBα and entails phosphorylation on Ser-32 and Ser-36 by the IKK complex, followed by subsequent ubiquitination and degradation via the 26S proteasome pathway. The less-characterized, atypical pathway of NF-κB induction involves tyrosine phosphorylation of IκBα. In contrast to serine phosphorylation of IκBα, tyrosine phosphorylation of IκBα at Tyr-42 generally does not invoke its ubiqutin-mediated degradation by the proteasome [6,7]. Instead, NF-κB activation by tyrosine phosphorylation of IκBα relies on a poorly defined mechanism whereby phospho-IκBα dissociates from NF-κB [8].

Our fundamental understanding of the interplay between tyrosine phosphorylation and NF-κB activation is principally derived from studies employing pervanadate, a phosphotyrosine phosphatase inhibitor. Pervanadate (PV) irreversibly inhibits tyrosine phosphatases while also inducing cellular oxidation, presumably due to phospho-tyrosine dependent activation of the cellular NADPH oxidases [9,10]. The pro-oxidant effects of PV are likely to have a stimulatory effect on protein tyrosine kinases (PTKs) because redox-sensitive mechanisms regulate the activation of cellular PTKs [11]. Hence, based on the cellular effects of PV, the plausible mechanisms by which PV activates NF-κB are two-fold: inhibition of protein tyrosine phosphatases and activation of protein tyrosine kinases. In support of this concept, an elegant study by Fan *et al.* demonstrated that c-Src-dependent tyrosine phosphorylation of IκBα and subsequent activation of NF-κB is contingent on intracellular H_2_O_2_ [12]. As added proof that hyperoxic conditions underlie the phospho-tyrosine-dependent mechanism of NF-κB induction, PV-induced NF-κB signaling mechanisms closely mimic those observed during hypoxia/reoxygenation, ischemia/reperfusion, and stimulation with growth factors [6,7,13,14,15,16,17]. Despite advances in our understanding of the interplay between redox mechanisms and phospho-tyrosine-dependent activation of NF-κB, the physiological significance of this redox-sensitive mechanism of NF-κB induction remains largely ill-defined.

Recent studies regarding “bortezomib resistance” have highlighted the biological significance of mechanisms of NF-κB induction, which are resistant to proteasome inhibition [18,19]. Hence, the *in vivo* relevance of the phospho-tyrosine-dependent mechanism of NF-κB induction may ultimately be defined due to its distinction as a proteasome-independent mechanism of NF-κB activation. Based on this prevision, we sought to investigate how proteasome inhibition affects facets of phospho-tyrosine-dependent NF-κB signaling, both related and unrelated to tyrosine phosphorylation of IκBα.

While considerable attention has been paid to the absence of proteasomal regulation of tyrosine phosphorylated IκBα, there is a paucity of information regarding proteasomal regulation of signaling events distinct from tyrosine phosphorylation of IκBα. Further, this includes an incomplete understanding of the role for ubiquitin-like modifiers, such as NEDD8 and SUMO, in the signaling events of the atypical NF-κB pathway. To delineate unexplored roles for the ubiquitin-proteasome pathway in the phospho-tyrosine dependent mechanism of NF-κB induction, we employed the proteasome inhibitor, Aclacinomycin, and the phosphotyrosine phosphatase inhibitor, pervanadate. Results from these studies demonstrate that phospho-IκBα (Tyr-42) is not subject to proteasomal degradation in a murine stromal epithelial cell line, confirming results previously reported in HeLa and Jurkat cell lines [7,12]. Correspondingly, proteasome inhibition had no discernable effect on the key signaling intermediaries—Src and ERK1/2—involved in the phospho-tyrosine mechanisms regulating PV-mediated activation of NF-κB. Consistent with previous reports, a significant redox imbalance leading to the activation of tyrosine kinases, as occurs with Pervanadate, is required for the induction of NF-κB in this cell type. Strikingly, our studies demonstrate that proteasome inhibition can potentiate oxidative stress associated with PV-stimulation; however, the cellular implications for this increase in intracellular oxidation remain to be delineated. In particular, this study highlights a regulatory mechanism underlying the inhibition of tyrosine phosphatases, a concomitant activation of tyrosine kinases accompanying cellular oxidation, and a significant role for proteasome inhibition in the potentiation of these responses.

## 2. Results

### 2.1. Pervanadate Stimulation Induces Tyrosine Phosphorylation of IκBα But Not Its Proteolytic Degradation

**Figure 1:**
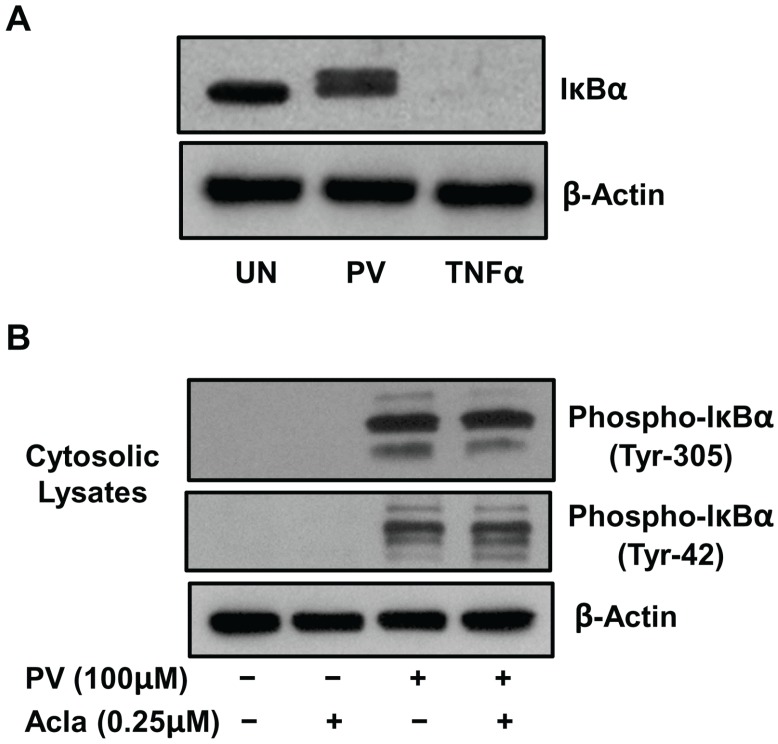
Pervanadate (PV) stimulation induces tyrosine phosphorylation of IκBα but not its proteolytic degradation. (**A**) ILU-18 cells were either left untreated or treated with Pervanadate (100 μM) or TNF-α (20 ng/mL) for 20 min. At the end of treatment, cytosolic lysates were obtained and 30μg protein from each lysate was resolved by sodium dodecyl sulfate polyacrylamide gel electrophoresis (SDS-PAGE). Resolved proteins were detected by Western blotting using antibody to nuclear factor of kappa light polypeptide gene enhancer in B-cells inhibitor, alpha (IκBα) and enhanced chemiluminescence (ECL). The blot was stripped and re-probed with antibody to β-actin to ensure equal protein loading. (**B**) ILU-18 cells were treated with PV (100 μM) for 20 min, with or without prior treatment with Aclacinomycin [Acla] (0.25 μM) for 2 h. At the end of incubation, cells were washed and cytosolic lysates prepared. As controls, cell lysates were made from ILU-18 cells either left untreated or treated for 140 min with 0.25 μM Aclacinomycin alone. Lysates, equalized for 30 μg protein, were resolved using SDS-PAGE, followed by Western blotting using a phospho-specific antibody recognizing IκBα (Tyr-42) or (Tyr-305). After stripping, the blot was re-probed with antibody to β-actin to demonstrate equal protein loading.

TNFα-mediated activation of NF-κB induction has been demonstrated to invoke serine phosphorylation of the inhibitory IκB proteins followed by ubiquitination and degradation via the 26S proteasome pathway [5]. In contrast, NF-κB activation by pervanadate involves tyrosine phosphorylation of IκBα and is not contigent upon proteasomal degradation of IκBα [6,7]. To test whether PV-mediated activation of NF-κB occurs by a proteasomal-independent mechanism in a murine stromal cell line, we subjected ILU-18 cells to short-term activation with TNFα or PV and then tested cytosolic lysates by immunoblotting with an antibody recognizing IκBα. While IκBα is no longer detected in response to TNFα treatment, IκBα remains in the cytosol following short-term PV treatment, indicating absence of IκBα degradation in PV-induced NF-κB (Figure 1A).

To elucidate whether PV-mediated NF-κB activation occurs by a mechanism involving tyrosine phosphorylation, we next assessed if both in the absence and presence of proteasome inhibitor (Aclacinomycin), IκBα undergoes tyrosine phosphorylation [20]. By employing immunoblotting with an antibody recognizing phospho-IκBα (Tyr42 or Tyr305), we now demonstrate that PV induces tyrosine phosphorylation of IκBα at Tyr-42 and Tyr305, both in the absence and presence of proteasome inhibitor (Figure 1B). Overall, these results demonstrate that in response to PV stimulation, tyrosine phosphorylation of IκBα occurs, independently of proteasome catalytic function.

### 2.2. Pretreatment with Inhibitors of Src and MEK but not p38, PI3K, JNK and IKK Complex Interfere with PV-Induced NF-κB Activity

**Figure 2:**
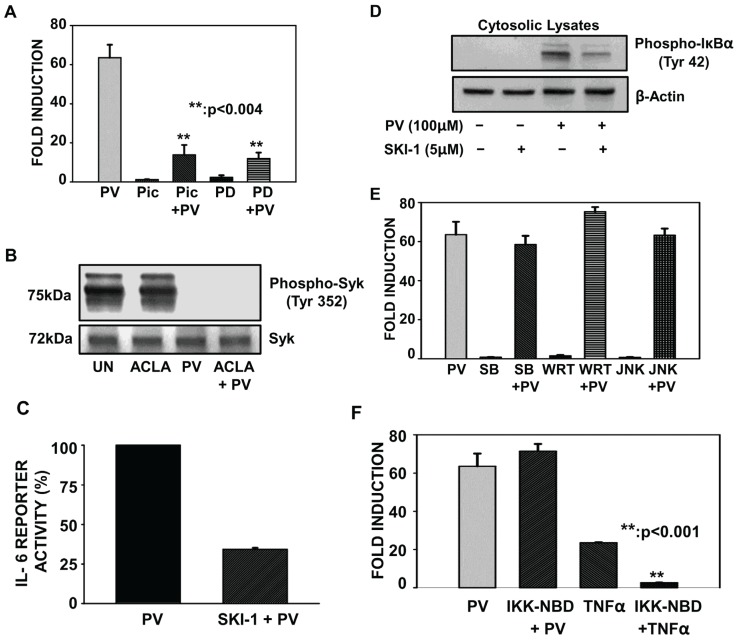
Pretreatment with inhibitors of Src and mitogen-activated protein kinase kinase (MEK) but not p38, PI3K, c-Jun N-terminal kinase (JNK) and IkappaB kinase (IKK) complex interfere with PV-induced NF-κB activity. (**A**) ILU-18 cells were pretreated with 75 µM Piceatannol (Pic.) for 45 min or 20 µM PD98059 (PD.) for 30 min followed by activation with 100 µM pervanadate for 18 h. Cells treated with 100 µM pervanadate, 75 µM Piceatannol, or 20 µM PD98059 for 18 h, served as controls. At the end of 18 h, lysates were obtained and luciferase activity was determined employing the Promega luciferase assay kit. Fold induction represents a ratio of luciferase activity obtained in treatment-induced to that of untreated cells. Data represents mean ± standard error obtained from four independent experiments, following normalization. ** Denotes significant difference at *p* < 0.004, between treatment groups. (**B**) ILU-18 cells were treated with PV (100 μM) for 20 min, with or without prior treatment with Aclacinomycin (0.25 μM) for 2 h. At the end of incubation, cells were washed and cytosolic lysates prepared. As controls, cell lysates were made from ILU-18 cells left untreated or treated 140 min with 0.25 μM Aclacinomycin alone. Lysates, equalized for 30 μg protein, were resolved using SDS-PAGE, followed by Western blotting using antibodies for phospho-Syk (Tyr-352) and, as a control, Syk. (**C**) ILU-18 cells were treated with 100 µM pervanadate for 18 h, with or without pretreatment for 6 h with 2 µM Src Kinase Inhibitor I (SKI-1). As controls, cells were either left untreated or subjected to treatment with 2 µM Src Kinase Inhibitor I for 24 h [25]. As described in (A), a luciferase assay was performed. Normalized data are presented as % activity with fold induction values from PV-treated cells set at 100%. Data represents mean ± S.E. from duplicates derived from two independent experiments. (**D**) ILU-18 cells were treated with 100 µM pervanadate for 20 min, with or without pretreatment for 4 h with 5 µM Src Kinase Inhibitor I (SKI-1). At the end of incubation, cells were washed and cytosolic lysates prepared. As controls, cell lysates were made from ILU-18 cells left untreated or treated 260 min with 5 µM Src Kinase Inhibitor I alone. Lysates, equalized for 30 μg protein, were resolved using SDS-PAGE, followed by Western blotting using a phospho-specific antibody recognizing IκBα (Tyr-42). The blot was stripped and re-probed with antibody to β-actin to ensure equal protein loading. (**E**) ILU-18 cells were treated with 100 µM pervanadate, 100nM SB23580 (SB.—p38 Kinase inhibitor), 10 µM Wortmannin (WRT—PI3K inhibitor), or 40 nM JNK Inhibitor for 18 h. ILU-18 cells were also pretreated for 1 h with 40 nM JNK Inhibitor or for 30 min with either 100nM SB23580 or 10 µM Wortmannin followed by activation with 100 µM pervanadate for 18 h. Fold induction was determined as in (A). Data represents mean ± S.E. from four independent experiments. (**F**) ILU-18 cells were either pretreated with 10 µM IKK-NBD for 1 h or left untreated. After 1 h, cells were either treated with 100 µM PV or 20 ng/mL TNFα for 18 h, yielding the following treatments: Untreated, PV, IKK-NBD+PV, TNFα, IKK-NBD+TNFα. Fold induction was determined as in (A). Data represents mean ± S.E. from duplicates derived from two independent experiments. ** Denotes significant difference at *p* < 0.001, between treatment groups.

Pervanadate mediates both the coordinated inhibition of cellular tyrosine phosphatases as well as the activation of cellular tyrosine kinases. Kinase families, which are prone to activation by PV, include the Src and Syk family of tyrosine kinases and the family of serine/threonine specific kinases, MAPK [12,15,21,22]. To delineate if these kinases are involved in NF-κB-dependent transcription induced by PV, we employed a luciferase reporter assay, which had previously been shown to be dependent on PV-mediated NF-κB activity. Pretreatment with Piceatannol, the resveratrol metabolite and tyrosine kinase inhibitor, resulted in a significant reduction in PV-induced NF-κB-dependent luciferase activity, thus indicating an important role for the Src/Syk family of tyrosine kinases in PV-mediated, NF-κB-dependent luciferase expression (Figure 2A). Furthermore, the mitogen-activated protein kinase kinase (MEK) inhibitor, PD98059, substantially interfered with PV-mediated NF-κB activity, indicating that MEK also contributes to pervanadate-mediated NF-κB activation (Figure 2A).

**Figure 3:**
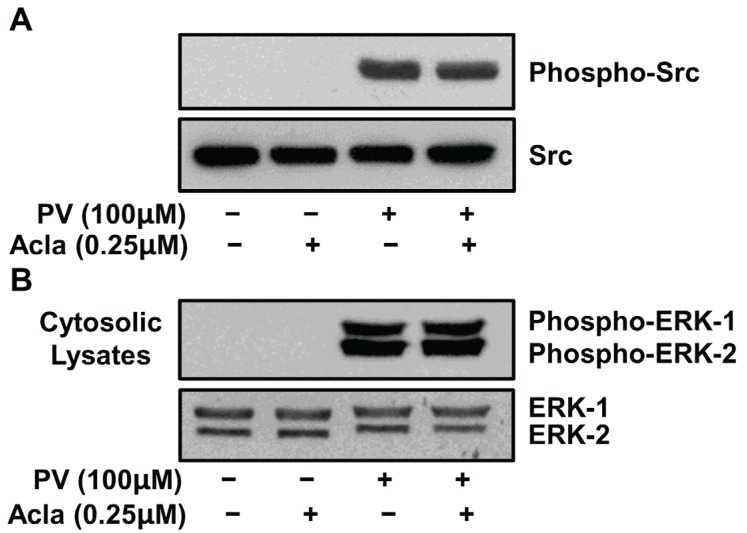
Proteasome inhibition does not impact PV-mediated activation of Src or MEK kinases. ILU-18 cells were treated with PV (100 μM) for 20 min, with or without prior treatment with Aclacinomycin (0.25 μM) for 2 h. At the end of incubation, cells were washed and cytosolic lysates prepared. As controls, cell lysates were made from ILU-18 cells left untreated or treated with 0.25 μM Aclacinomycin alone for 140 min. Lysates, equalized for 30 μg protein, were resolved using SDS-PAGE, followed by Western blotting using antibodies to phospho-Src (Tyr-416) and Src (**A**); or antibodies to phospho-p44/p42 ERK1/2 (Thr-202/Tyr-204) and ERK1/2 (**B**).

To elucidate which family of tyrosine kinases is principally involved in PV-dependent NF-κB activation, we subjected ILU-18 cells to short-term activation with pervanadate (20 min) and then examined Syk and phospho-Syk by immunoblotting with antibodies recognizing Syk & tyrosine-phosphorylated Syk. Though Syk was detected, tyrosine phosphorylation of Syk was not observed following PV treatment in ILU-18 cells (Figure 2B). In contrast, we confirmed that c-Src is indeed phosphorylated following PV stimulation in this cell line by employing an antibody to phospho-Src (Figure 3). Given the PV-mediated phosphorylation of c-Src, we sought to determine if Src kinase was necessary in PV-dependent NF-κB activity. Employing a selective inhibitor of Src, we pretreated cells with Src Kinase Inhibitor I, before subjecting them to PV-stimulation. As depicted in Figure 2C, pretreatment with Src Kinase Inhibitor I significantly inhibited PV-induced NF-κB activity, thus demonstrating the involvement of Src kinase in PV-induced activation of NF-κB. Since c-Src kinase has been implicated in the tyrosine phosphorylation of IκBα, we evaluated if in the ILU-18 cell line, Src kinase plays a role in the tyrosine phosphorylation of IκBα at tyrosine 42. Pretreatment with Src Kinase Inhibitor I abrogated PV-dependent, tyrosine phosphorylation of IκBα (Tyr-42), as determined by immunoblotting with an antibody recognizing phospho-IκBα (Tyr-42) (Figure 2D). Taken together, these results demonstrate that c-Src contributes to PV-mediated NF-κB activation by phosphorylating IκBα at tyrosine 42.

As JNK, p38, and PI3K subfamilies have been connected with stress conditions conducive for inactivation of tyrosine phosphatases [23], we next tested their involvement in PV-mediated NF-κB signaling by employing inhibitors to the kinase activity of each of these pathways (Figure 2E). Unlike MEK and the Src family kinases, the involvement of JNK, p38, and PI3K activity was found not to be critical in pervanadate-mediated NF-κB activation. As IKK complex activation following activation of PKD has been demonstrated to be necessary in H_2_O_2_-induced NF-κB responses in epithelial cells [24], we next tested the role of IKK in PV-induced NF-κB activity. Employing a cell permeable IKK Nemo-binding peptide (IKK-NBD) as an inhibitor of IKK complex formation, we demonstrate that pretreatment with IKK-NBD did not inhibit pervanadate-mediated luciferase expression, indicating that luciferase expression is independent of the IKK complex. TNFα induced IL-6 expression was, however, dependent on IKK (Figure 2F). Thus, pretreatment with Src and MEK inhibitors, but not JNK, p38, PI3K, and IKK complex inhibitors, modulated pervanadate-induced luciferase expression, providing evidence that Src and MEK kinases are key signaling intermediaries in PV-mediated NF-κB induction.

### 2.3. Proteasome Inhibition Does Not Alter PV-Mediated Activation of Src or MEK Kinases

As PV represents a proteasome-independent mechanism of NF-κB induction, we assessed the effect of proteasome inhibition on the signaling pathways identified in PV-induced NF-κB activity, e.g., Src and MEK kinases. To determine the effect of proteasome inhibition on the activation of the Src and ERK 1/2 kinases, we subjected ILU-18 cells to short-term activation with PV, both in the absence or presence of proteasome inhibitor, and then tested cytosolic lysates by immunoblotting with antibodies recognizing phosphorylated c-Src and ERK 1/2. As depicted in Figure 3, both in the absence or presence of proteasome inhibitor, pervanadate induced the phosphorylation of c-Src and ERK 1/2. Thus, loss in proteasomal catalytic activity does not interfere with the phospho-tyrosine dependent mechanisms that regulate PV-mediated induction of NF-κB.

### 2.4. PV-Mediated Activation of NF-κB Involves Oxidative Stress

**Figure 4:**
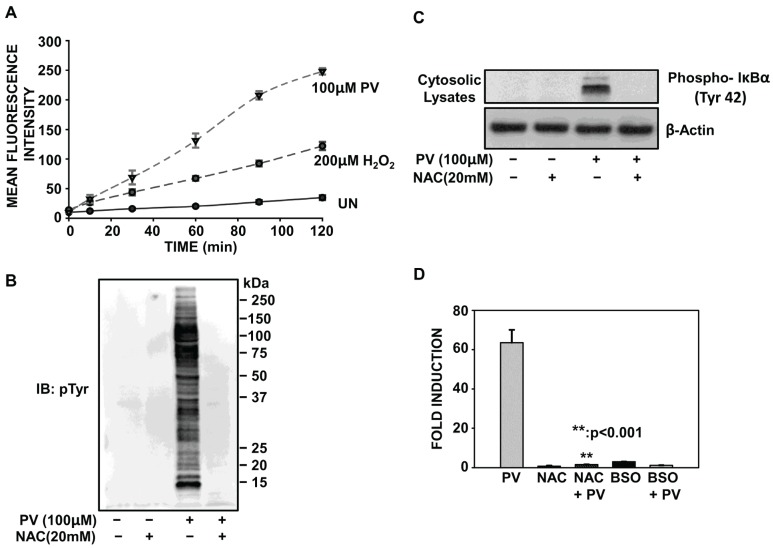
PV-mediated activation of NF-κB involves oxidative stress. (**A**) ILU-18 cells were washed and briefly incubated in 1X Hank’s Balanced Salt Solution (HBSS), prior to incubation with 10 μM H_2_DCF-DA for 30 min at 37 °C in the dark. At the end of the incubation, cells were washed and resuspended in 1X HBSS. Intracellular reactive oxygen species (ROS) generation was detected following addition of PV (100 µM) or H_2_O_2_ (200 µM), as described in the methods section. (**B**) ILU-18 cells were either untreated or treated with NAC (20 mM) for 2 h. After media replacement, cells were selectively treated with PV (100 μM) for 20 min. At the end of incubation, cells were washed and cytosolic lysates prepared. As controls, cell lysates were made from ILU-18 cells left untreated or treated with 20 mM NAC alone for 140 min. Lysates, equalized for 30 μg protein, were resolved using SDS-PAGE, followed by Western blotting using an antibody specific to Phospho-Tyrosine residues. Molecular weights derived from standards are indicated in kDa. (**C**) Cytosolic lysates (30 µg), obtained as in (B), were resolved using SDS-PAGE, followed by Western blotting using a phospho-specific antibody recognizing IκBα (Tyr-42). After stripping, the blot was re-probed with antibody to β-actin to demonstrate equal protein loading. (**D**) ILU-18 cells were either untreated or treated with NAC (20 mM) for 2 h. After media replacement, cells were selectively treated with PV (100 μM) for 18 h. Additionally, ILU-18 cells were treated with 100 μM H_2_O_2_ for 18 h or subjected to treatment with 200 μM BSO for 24 h. Lysates obtained were evaluated for luciferase acitivity, as described previously. Data obtained from at least four independent experiments are presented; values represent data means ± standard error. ** Denotes significant difference at *p* < 0.001, between treatment groups.

While the precise mechanisms responsible for PV-mediated induction of NF-κB remain to be fully explored, it is known that the redox imbalance accompanying pervanadate stimulation plays a significant role in both tyrosine phosphorylation of IκBα and NF-κB transcriptional activity [12]. To assess if PV acts as a potent pro-oxidant in ILU-18 cells, we measured the generation of reactive oxygen species (ROS) by employing the oxidant-sensing fluorescent probe, H_2_DCF-DA. Compared to the induction of ROS by the exogenous addition of H_2_O_2_, PV treatment was more potent at inducing intracellular DCF fluorescence (Figure 4A). Thus, exposure to PV induces ROS generation, as primarily reported in Jurkat and Ramos cells [26].

To ascertain the functional significance of redox imbalance, we determined if cellular tyrosine phosphorylation induced by PV was dependent upon intracellular oxidation. By measuring oxidative stress with the H_2_DCF-DA probe, we determined that pretreatment of ILU-18 cells with the antioxidant, *N*-acetylcysteine (NAC), significantly impaired the pro-oxidant effects of PV [25]. Based on this observation, we pretreated ILU-18 cells with NAC to boost nonenzymatic detoxification of reactive electrophiles and free radicals and then measured PV-dependent phospho-tyrosine accumulation by immunoblotting with an anti-phosphotyrosine antibody. As depicted in Figure 4B, pretreatment with NAC inhibited the induction of cellular tyrosine phosphorylation by PV. Additionally, NAC pretreatment blocked the tyrosine phosphorylation of IκBα (Tyr-42) accompanying PV-stimulation (Figure 4C). Taken together, these results demonstrate that the generation of ROS and subsequent intracellular oxidation by PV plays a prominent role in tyrosine phosphorylation of cellular proteins, including IκBα.

To further support the contention that NF-κB activation by PV occurs as a result of oxidative stress, we evaluated the effect of NAC pretreatment on PV-mediated NF-κB activity. As expected, the ROS scavenging effects of NAC offset Pervanadate-mediated oxidative stress, resulting in levels of luciferase activity on a par with background levels (Figure 4D). This reversal by NAC demonstrates that Pervanadate is inducing luciferase expression by the generation of ROS.

Having established that Pervanadate induces NF-κB activation by oxidative stress, we next tested whether perturbations in the redox balance were sufficient to induce NF-κB activity. For this, NF-κB-dependent luciferase expression was analyzed using Buthionine-sulfoximine (BSO) treatment. BSO mediates its pro-oxidant effects by inhibiting the enzyme responsible for synthesis of the primary intracellular thiol and ROS scavenger, glutathione (GSH). BSO treatment of ILU-18 cells for 24 h resulted in only a two-fold induction of luciferase activity (Figure 4D). These BSO results would likely have been higher if BSO was co-administered with a pro-oxidant, thereby more potently disrupting the redox balance and activating NF-κB. Similarly, H_2_O_2_ did not induce NF-κB-dependent luciferase activity (Figure 4D). This finding is consistent with our observations that in ILU-18 cells, H_2_O_2_ was neither a potent pro-oxidant (Figure 4A) nor an effective inducer of cellular tyrosine phosphorylation [25]. Collectively, these results indicate that a significant redox imbalance leading to the activation of tyrosine kinases, as occurs with Pervanadate, is required for the induction of NF-κB in this cell type.

### 2.5. Proteasome Inhibition Potentiates Oxidative Stress Associated with PV-Stimulation

**Figure 5:**
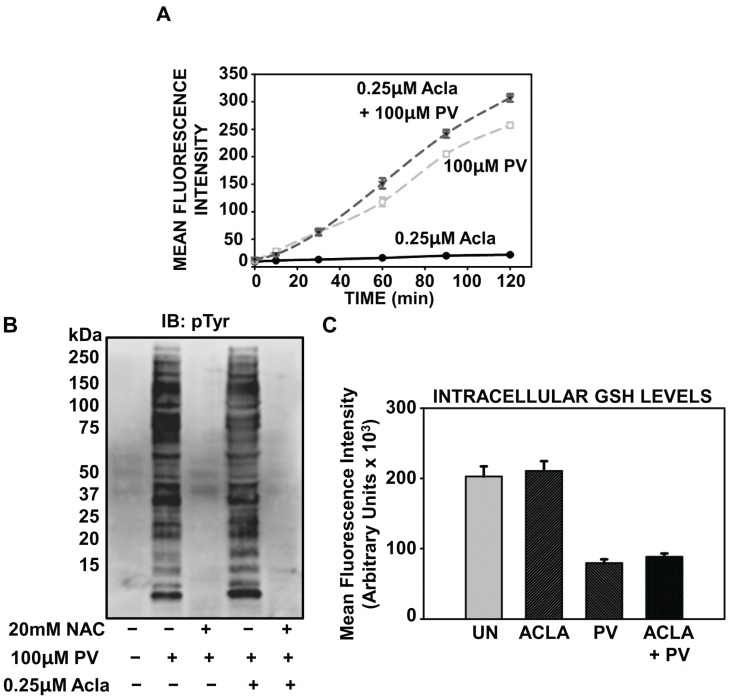
Proteasome inhibition potentiates oxidative stress associated with PV-stimulation. **(A**) ILU-18 cells were either left untreated or treated with 0.25 µM Acla for 2 h. Following treatment with proteasome inhibitor, cells were washed and briefly incubated in 1X HBSS, prior to incubation with 10 μM H_2_DCF-DA for 30 min at 37 °C in the dark. At the end of the incubation, cells were washed and resuspended in 1X HBSS. Intracellular oxidation was detected following addition of PV (100 µM), as described in the methods section. (**B**) ILU-18 cells were either left untreated or pretreated with 0.25 µM Acla for 2 h, in the presence or absence of 20 mM NAC. Media was then replaced and cells were selectively treated with PV (100 µM) for 20 min. At the end of incubation, cells were washed and cytosolic lysates prepared. Lysates, equalized for 30 μg protein, were resolved using SDS-PAGE, followed by Western blotting using an antibody specific to Phospho-Tyrosine residues. Molecular weights derived from standards are indicated in kDa. (**C**) ILU-18 cells were treated with 100 µM pervanadate for 18 h, with or without pretreatment for 2 h with 0.25 µM Aclacinomycin. As controls, cells were either left untreated or subjected to treatment with 0.25 µM Aclacinomycin for 20 h. At the end of treatment, cells were lysed; 2 µL of MCB and GST were added to each sample and incubated at 37 °C for 15 min. Fluorescence was measured at an excitation wavelength of 340 nm and emission wavelength of 465 nm. Data obtained from duplicates of at least two independent experiments are presented; values represent mean fluorescence intensity (MFI) ± standard error.

While proteasome inhibition has no effect on the phospho-tyrosine dependent mechanisms that regulate PV-mediated induction of NF-κB (Figure 3), it remains unclear how proteasome inhibition impacts the intracellular redox balance, which is also critically necessary for PV-mediated induction of NF-κB. By measuring generation of ROS with the H_2_DCF-DA probe, we observed that pretreatment with proteasome inhibitors potentiated oxidative stress generated by pervanadate stimulation (Figure 5A). ILU-18 cells treated with proteasome inhibitor alone induced intracellular oxidation near background levels, thereby affirming that proteasome inhibition does not independently induce oxidative stress (Figure 5A). Despite the amplification in intracellular ROS generation, pretreatment with proteasome inhibitor did not intensify cellular tyrosine phosphorylation accompanying PV treatment (Figure 5B). Additionally, pretreatment with proteasome inhibitor did not interfere with redox-dependent tyrosine phosphorylation induced by PV (Figure 5B). Thus, while loss in proteasomal catalytic activity enhances PV-mediated oxidative stress, this effect appears to have no discernable impact on redox-dependent tyrosine phosphorylation by PV.

For an assessment of the long-term implications of proteasome inhibition on PV-induced ROS generation, we measured intracellular glutathione (GSH) following 18 h of PV stimulation. As GSH participates in the enzymatic conversion of H_2_O_2_ to H_2_O, depletion of this antioxidant is indicative of oxidative stress within the cell. Both in the absence and presence of proteasome inhibitor, GSH levels declined significantly after 18 h exposure to PV. Yet, despite pretreatment with proteasome inhibitor, PV-associated loss in functional GSH was nearly equivalent; in other words, proteasomal inhibition did not result in an elevation in GSH depletion (Figure 5C). In agreement with ROS data, treatment with proteasome inhibitor alone had no effect on GSH levels (Figure 5C), indicating that loss in proteasomal catalytic activity alone is not sufficient to induce intracellular oxidation in ILU-18 cells. Overall, these results show for the first time that proteasome inhibition can potentiate oxidative stress associated with PV-stimulation; however, the cellular source and implications for this increase in intracellular oxidation have to yet to be determined.

## 3. Discussion

For a subset of NF-κB signaling pathways, proteasome inhibition is ineffective at blocking NF-κB induction because these pathways rely on proteasome-independent mechanisms for releasing NF-κB from its inhibitor IκBα. For instance, the atypical pathway of NF-κB induction entails upstream signaling mechanisms, which culminate in tyrosine phosphorylation of IκBα and subsequent dissociation from NF-κB without the involvement of the proteasome. Considerable attention has been paid to the absence of proteasome-mediated degradation of IκBα within this subset of NF-κB signaling pathways; however, the effect of proteasome inhibition on additional upstream signaling events in these pathways remains unexplored. For this reason, we delineated the effect of proteasome inhibition on facets of phospho-tyrosine-dependent NF-κB induction, both related and unrelated to tyrosine phosphorylation of IκBα.

We demonstrate in a murine bone marrow-derived stromal cell line that, upon short-term activation with PV, cytosolic IκBα undergoes tyrosine phosphorylation but not proteasome-mediated degradation—in keeping with data reported from previous studies employing HeLa and Jurkat cell lines [7,12]. Additionally, as in HeLa and Jurkat cell lines, phosphorylation of IκBα at Tyr-42 is regulated by the tyrosine kinase, c-Src. Based on immunoblotting, we demonstrate that c-Src assumes its active form, regardless of proteasome inhibition. Since these results analyzed Src kinase activation following short-term exposure to PV, we cannot exclude the possibility that after prolonged exposure to PV, a discrepancy in Src kinase activity may ensue from the combined treatment of proteasome inhibitor and PV, as compared to PV treatment alone. This stems from studies describing the active form of Src as a preferential target for degradation by the proteasome [27,28]. The intriguing possibility that proteasome down-regulates PV-dependent Src activity introduces the idea of the proteasome as a key negative regulator of signaling associated with the atypical NF-κB pathway. At the present time, there is no evidence in the literature, which either supports or refutes this hypothesis. However, stabilization of Src by the chaperone, Hsp 90, has recently been shown to be an essential mechanism in the Src-regulated phosphorylation of IκBα^Y42^ accompanying PV stimulation [29]. This emphasizes that mechanisms associated with the regulation of Src have a profound impact on PV-mediated induction of NF-κB. Accordingly, Src represents a “lynch pin” in the induction of NF-κB by PV, and as such, constitutes a prime target for negative regulators of the atypical NF-κB pathway.

Whereas c-Src kinase phosphorylates IκBα at Tyr-42 [12], the tyrosine kinase, c-Abl, has been implicated in the phosphorylation of IκBα at Tyr-305 [30]. The precise role of phospho-IκBα (Tyr-305) in the atypical NF-κB signaling pathway remains elusive because the stimuli generally associated with atypical NF-κB responses have yet to be vetted as inducers of phospho-IκBα (Tyr-305). Thus far, overexpression of either a constitutively-active c-Abl protein or Hepatitis C virus nonstructural protein 5A (NS5A) has been conclusively shown to induce the tyrosine phosphorylation of IκBα at Tyr-305 [30,31]. Due to discrepancies in the findings between these studies, tyrosine phosphorylation of IκBα^Y305^ appears to modulate both its stability as well as its ability to simultaneously undergo tyrosine phosphorylation at Tyr-42, in a stimulus-dependent manner. To our knowledge, PV has not been characterized as an inducer of phospho-IκBα (Tyr-305) but as we demonstrate here, PV can indeed induce tyrosine phosphorylation of IκBα at both Tyr-42 and Tyr-305 residues. Without rigorous, biochemical experimentation, we are unable to definitively elucidate whether PV induces pools of IκBα which are uniquely phosphorylated at each tyrosine residue (Tyr-42 or Tyr-305), dually modified at each tyrosine residue (Tyr-42 and Tyr-305), or a combination of both. Given that Tyr-42/-305 phospho-IκBα has been demonstrated to undergo calpain-mediated degradation in NS5A overexpressing cells, it is noteworthy that PV-induced phospho-IκBα does not undergo any degradation in ILU-18 cells. Further, pretreatment with the calpain inhibitor, E64D, prior to PV-treatment did not interfere with NF-κB activity [25]. Nevertheless, our demonstration that PV is capable of inducing tyrosine phosphorylation of IκBα at both tyrosine residues provides the assurance that PV can be used to evaluate the crosstalk not only between these two tyrosine phosphorylation sites but also the potential crosstalk between tyrosine phosphorylation and sumoylation.

Strikingly, modification of IκBα by either tyrosine phosphorylation or sumoylation confers similar outcomes in terms of the stability. Since IκBα undergoes modification by SUMO-1 at the same lysine residue targeted for ubiquitination (K21), sumoylated IκBα is resistant to ubiquitin-mediated degradation by the proteasome [32]. Accordingly, modification of IκBα by SUMO-1 has emerged as the prototypical example of the stabilizing effect of sumoylation. Similarly, tyrosine phosphorylation has been found to protect IκBα from the cytokine-induced serine phosphorylation which precedes ubiquitin-mediated degradation by the proteasome [33]. Despite the overlap in function between these two modifications, the possibility that IκBα is dually modified by SUMO-1 and tyrosine phosphorylation remains unexplored.

Finally, the objective of this study was to understand the effect of proteasome inhibition on facets of phospho-tyrosine-dependent NF-κB signaling, both related and unrelated to tyrosine phosphorylation of IκBα. While proteasome inhibition had no discernable effect on the early signaling events accompanying PV-mediated NF-κB induction, proteasome inhibition did potentiate oxidative stress associated with PV-stimulation. The cellular implications for this increase in intracellular oxidation are uncertain in this cell type, however, there is mounting evidence that proteasome inhibition, in combination with oxidative stress, has appreciable effects on cellular viability, clearance of oxidatively damaged proteins, detoxification capacity of antioxidant enzymes, and inflammation in other cell types [34,35,36,37]. In neuronal cells, both acute and chronic inhibition of proteasome activity alters mitochondria homeostasis, resulting in overproduction of reactive oxygen species (ROS) by dysfunctional mitochondria [37,38]. Contrary to these reports, in our system, proteasome inhibition alone did not induce an increase in ROS. Instead, proteasome inhibition had an additive effect on cellular oxidation induced by PV. We investigated the possibility that the combination of proteasome inhibition and oxidative stress unduly stressed detoxification capacity of GSH but despite pretreatment with proteasome inhibitor, PV-associated loss in functional GSH was nearly equivalent. However, a potential effect of proteasome inhibition on additional antioxidants, such as catalase and superoxide dismutase remains to be tested. In fact, this possibility is supported by a recent study delineating the effect of pretreatment with proteasome inhibitor on liver ischemia/reperfusion (I/R). In this study, Alexandrova *et al.* demonstrated that compared to I/R alone, the combination of proteasome inhibition and I/R disproportionately stressed the activities of the antioxidant enzymes, catalase and superoxide dismutase [39].

In summary, the results presented here reiterate that early signaling events associated with atypical pathway of NF-κB induction are proteasome-independent. While mechanisms and biological significance of IκBα tyrosine-phosphorylation in protein stability remain unclear, these studies highlight the potential for an intriguing interplay between redox regulation, tyrosine phosphorylation, and the atypical mechanism of NF-κB induction.

## 4. Experimental Section

### 4.1. Antibodies & Reagents

Phospho-Tyrosine, phospho-p38 MAPK (Thr-180/Tyr-182), phospho-p44/p42 MAPK (Thr-202/Tyr-204), Src, and phospho-Src (Tyr-416) antibodies were obtained from Cell Signaling Technology (Danvers, MA, USA). Antibodies for phospho-specific IκBα (Tyr-42) and (Tyr-305) were purchased from ECM Biosciences (Versailles, KY, USA). All other antibodies were from Santa Cruz Biotechnology (Santa Cruz, CA, USA). Anti-IgG coupled to horseradish peroxidase and enhanced Chemiluminescence (ECL) reagents were from ThermoScientific (Rockford, IL, USA). All fine chemicals, unless otherwise mentioned, were obtained from Sigma Chemical Company (St. Louis, MO, USA). Electrophoresis supplies and Molecular weight standards were from BioRad (Hercules, CA, USA). Aclacinomycin, Lactacystin, SB23580, Wortmannin, and JNK Inhibitor II, and PD98059 were from EMD Millipore (Bilerica, MA, USA). Murine TNFα was purchased from R&D Systems (Minneapolis, MN, USA). 2,7-dichlorodihydrofluorescein diacetate (H_2_DCF-DA) was purchased from Life Technologies (Grand Island, NY, USA). Piceatannol was obtained from Enzo life sciences (Farmingdale, NY, USA).

### 4.2. Cell Culture

ILU-18 cells were maintained in RPMI supplemented with 2 mM glutamine, 100 units/mL penicillin, 100 µg/mL streptomycin, and 10% fetal bovine serum. This cell line was stably transfected with a murine IL-6 minigene designated ILU, containing the entire IL-6 promoter and first intron, including the 3′UTR. Derived from the bone marrow stromal cell line, ^+/+^LDA.11, ILU-18 cells were kindly provided by Dr. Charles O’Brien (UAMS, Little Rock, AR, USA).

### 4.3. Preparation of Cytosolic and Nuclear Extracts and Western Blotting

Cells were washed twice in cold phosphate-buffered saline, resuspended in cytosolic extraction buffer containing 10 mM Hepes, pH 7.8, 10 mM KCl, 2 mM MgCl_2_, 0.1 mM EDTA, 1 mM DTT, and protease inhibitor cocktail, and incubated on ice for 20 min. Nonidet P-40 was added to the cells to obtain a final concentration of 0.5%, followed by mixing and centrifugation at 12,000× *g* for 5 min at 4 °C. Supernatants, which corresponded to cytosolic extracts, were collected. Nuclear extracts were prepared by resuspending the pellet in nuclear extraction buffer containing 50 mM Hepes, pH 7.8, 50 mM KCl, 300 mM NaCl, 0.1 mM EDTA, 1 mM DTT, 10% glycerol, and protease inhibitor cocktail. Proteins were extracted by vortexing for 60 min at 4 °C and clarified by centrifugation at 14,000× *g* for 10 min. Supernatants which represented both cytosolic and nuclear extracts were collected and analyzed for protein content by Bio-Rad protein assay. Cell lysates equalized for protein were resolved by SDS-PAGE, transferred to nitrocellulose, immuno-blotted with specific antibodies, and detected using anti-IgG coupled to horseradish peroxidase (HRP) followed by ECL.

### 4.4. Measurement of Intracellular Oxidation Using H_2_DCF-DA

ILU-18 cells (1 × 10^6^/mL) were washed with 1X Hank’s Balanced Salt Solution (HBSS) before resuspending the cells in 1 mL HBSS and equilibrating at 37 °C for 15 min. At the end of equilibration, cells were treated with 10 μM H_2_DCF-DA and incubated further for 30 min at 37 °C in the dark. After washing the cells with 1X HBSS, cells were resuspended in 1 mL of 1X HBSS. ROS generation was monitored following addition of PV (100 μM) or H_2_O_2_ (200 μM) and detected using a LS-50 spectrofluorometer (Perkin Elmer Corporation, Norwalk, CT, USA) at excitation wavelength of 480 nm and emission wavelength of 525 nm.

### 4.5. Measurement of Intracellular GSH

Intracellular GSH levels were assayed as described previously [40]. Briefly, 100 μL of ice-cold lysis buffer was added to ILU-18 cells (1 × 10^6^), incubated on ice for 10 min, and centrifuged at 12,000× *g* for 10 min. To this preparation, 2 μL of 25 mM monochlorobimane (MCB) and 50 U/mL glutathione-*S*-transferase (GST) were added and incubated at 37 °C for 30 min. At the end of incubation, fluorescence was measured by employing a fluorescence plate reader (Perkin Elmer Corporation, Norwalk, CT, USA) with an excitation wavelength of 340 nm and emission wavelength of 465 nm. Values obtained in the absence of GST and MCB served as negative controls.

### 4.6. Luciferase Reporter Assay

ILU-18 cells, with or without pretreatment, were activated with pervanadate (100 μM) for 18 h at 37 °C. Additionally, ILU-18 cells were subjected to treatment with H_2_O_2_ (100 μM) for 18 h or 200 μM Buthionine-sulfoximine (BSO) for 24 h at 37 °C. Utilizing a Luciferase Reporter assay kit (Promega, Madison, WI, USA), lysates were prepared with Reporter lysis buffer by repeated freezing and thawing. Protein content was determined by Bio-Rad assay. Per the supplier’s protocol [41], cell extract (50 μg protein) was combined with 100 μL of the luciferase assay reagent at room temperature and analyzed by luminometer.

### 4.7. Statistical Analyses

Differences between means of the data generated in the study were analyzed using Student’s *t*-test. Differences were considered significant, if *p* < 0.05.

## 5. Conclusions

The results presented here demonstrate that while proteasome inhibition can potentiate oxidative stress associated with pervanadate stimulation, it fails to influence the induction of NF-κB by the atypical pathway. Thus, the regulation of IκBα and upstream signaling pathway intermediates in the atypical pathway of NF-κB induction, are also proteasome- independent.
